# From Mysore to Cambridge and back: The education of a groundnut breeder

**DOI:** 10.1002/ppp3.10450

**Published:** 2023-12-07

**Authors:** Tad Brown

**Affiliations:** Department of History and Philosophy of Science, University of Cambridge, Cambridge, UK

**Keywords:** agriculture, crop breeding, genetics, groundnuts, history of science

## Abstract

Studies that show how empire influenced the development of plant genetics add to the established history of genetics and 20th-century agricultural science. One approach to broadening this history is to consider the contributions of students studying abroad and their greater careers back home. Research agendas differed between and within institutions, much as they do today. This article explores the postgraduate education of an Indian groundnut breeder. It highlights the structural challenges faced by researchers at agricultural departments who sought promotion through education and examines the consequences for plant breeding and for farmers.

**Summary:**

- Through the biography of V.K. Badami, this article contributes to debates about genetics and plant breeding in the history of science. Badami, an accomplished breeder in the Mysore Department of Agriculture, took a leave to study genetics at the University of Cambridge. His professors nearly failed him, yet Badami's groundnut breeding experiments proved influential for Indian farmers as well as advances in crop science. This history adds to the thesis that academic genetics varied in support of professional plant breeding by comparing institutional expections between Mysore and Cambridge.
- The argument is developed by reading Badami's student records along with his groundnut breeding experiments in South India.
- The study connects the disciplinary history of genetics to plant breeding for the British empire.
- Badami's experience at Cambridge is indicative of the power relationship between academic genetics and imperial plant breeding in the early 20th century. In this case, his commitments as an Indian agricultural officer conflicted with the discipline’s devotion to quantitative analysis.

## 1 Introduction

Years ago, Daniel Kevles (1980) urged fellow historians to explore beyond the canon of principle figures in the historiography of genetics. A sizable literature has since expanded the topic to recognize other scientific contributors, including many plant breeders (Berry, 2021). The context for these works, which debate the influence of Mendelian genetics on plant breeding, has centered on Sweden (Müller-Wille, 2005; Roll-Hansen, 2000), France (Bonneuil, 2006; Gayon & Zallen, 1998), Germany (Harwood, 1997; Wieland, 2006), England (Berry, 2014; Charnley & Radick, 2009; Palladino, 1993), and the United States (Allen, 2000; Paul & Kimmelman, 1988). Agriculture proved to be a key site for the production of genetic knowledge, even if before 1930, the new theoretical framework resulted in incremental changes to 19th-century techniques—not a revolution in breeding practice (Harwood, 2015).

The same literature identified a split between academic geneticists and professional plant breeders in the early 20th century. For those in the commercial trade, the science of plant breeding was demonstrated by new varieties, whereas “method-conscious geneticists” found little for the advancement of Mendelian theory in outdoor tinkering and its random sports (Palladino, 1994, p. 417). For example, when biologist George Harrison Shull visited the famous plant breeder Luther Burbank, Shull expressed open skepticism about the scientific value of Burbank's work, calling his records “utterly useless” (Glass, 1980, p. 136). Biographical comparisons between national contexts, however, have revealed how breeders met with different levels of support from scientific institutions. Paolo Palladino (1994, p. 432) theorized that the reception of Mendelian genetics varied based on the political economy of agricultural research yet suggested the need to consider other contexts. This article reinforces Palladino's argument by showing how professional plant breeding conflicted with the academic study of plant genetics due to expectations about the nature of scientific work within the university.

Historians of science have, more generally, been occupied with broadening the geographical scope of the discipline and challenging the idea that European colonies were subjugated outposts where “peculiarly Western forms of knowledge” proved unimpeachable (Palladino & Worboys, 1993). Reference to a “center” and “periphery” of scientific knowledge production is now a dated feature of historical narration (Chakrabarti & Worboys, 2020). The challenge that persists is how to discuss cultural differences and power imbalances without insisting on a Eurocentric understanding of science (Chakrabarti, 2021; Sivasundaram, 2010). One key provocation has been to situate historical analysis on the circulation of “knowledge makers themselves” (Raj, 2013, p. 345). There is clearly a need for the historiography of genetics to include sites and experiences beyond those already studied. A focus on the greater careers of international students offers one approach.

Past presumptions about the location of scientific knowledge have caused remote experts within the imperial economy to go overlooked (for exceptions, Kumar, 2020; Ratcliff, 2016; Zimmerman, 1996). Nevertheless, scholarship on tropical crop breeding, and sugarcane in particular, has consistently refuted the idea that research in the colonies was of a subordinate nature (Galloway, 1996; Storey, 1997) A few historians of science have also recently begun to ask how agricultural experts dialogued with Mendelism in different contexts (Charnley, 2013; Soto Laveaga, 2018). Building on this strong foundation, I follow Barbara Kimmelman (1987) to detail what it was like for agricultural practitioners to study genetics in the early 20th century. Not only did Cambridge dons go overseas to investigate plant breeding, professional plant breeders from overseas came to Cambridge to study genetics.

This article highlights the pioneering work of Venkata Rao Krishnaji Badami (1888–ca. 1950).^1^ During his career, leading crop scientists recognized Badami's talents as a plant breeder (e.g., Russell, 1937, p. 198), but his work has since lapsed into obscurity among historians and the reading public. For example, press reports in the United States claim that a crop scientist at the University of Florida was first to hybridize groundnuts in 1928 (Haire, 2019). Successful hybrids by Badami (1922) predate that programme. The implications extend beyond groundnuts. Recovering the scientific work of unsung agronomists can help redress the canon of heroes in 20th-century agricultural history and its implications for discussions of modern crop breeding (Harwood, 2018; Soto Laveaga, 2018).

For breeders like Badami, who was stationed in the Mysore Agricultural Department, going abroad for postgraduate studies provided an opportunity for career advancement. To succeed, he had to conduct himself across disjointed fields, the field of plant genetics and the crop fields of South India. Accomplishments in either field did not readily translate to the other. Badami fulfilled his service to the department in the form of crop varieties, whereas the same breeder, to earn passing credit at Cambridge, was asked to produce quantitative analyses. This article shows that obtaining a doctorate enabled Badami to become the Economic Botanists of Mysore, yet his professional commitments to public plant breeding interferred with his academic studies.

## 2 Prelude to a Postgraduate

V.K. Badami was born and educated in Mysore, a princely state within British India. Badami belonged to a longstanding Maharathi military family. His father, educated in Mysore, served in various government offices, including the Vice Chairman of the Bangalore Municipality, and instilled into his sons an abiding interest in agriculture. As the youngest son, V.K. Badami committed to his studies and passed with distinction from the Coimbatore College of Agriculture before joining the Mysore Agriculture Department as Junior Assistant Botanist in 1913. He advanced to become the Assistant Principal of the Agricultural School at Hebbal and, in 1918, Senior Assistant Botanist for the department.^2^ This was Badami's position when he went overseas.

The Mysore government deputed Badami for postgraduate studies at the University of Cambridge in 1924, where he studied plant breeding and genetics under the direction of Sir Rowland Biffen (on Biffen, see Palladino, 1993; Charnley, 2016). Badami also studied with Professor R.C. Punnett (of the Punnett Square) and sugarcane breeder Dr. C.A. Barber, and while in Europe, he met with Herman Nilson-Ehle and Wilhelm Johannsen—all stock names in the history of genetics and plant breeding (see Berry, 2014; Müller-Wille, 2005). Not only was Badami relatively privileged in the social context of South India, the secondment granted him a host of notable contacts in the field of plant genetics.

**Figure 1:**
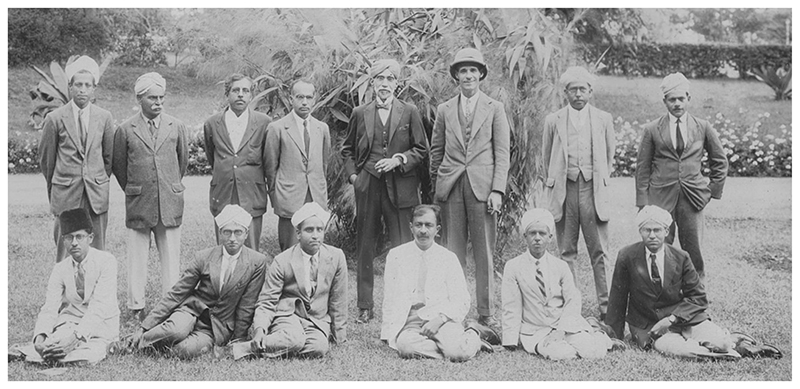
Badami, in white blazer, seated middle front with no head covering; Coleman stands behind him, wearing a pith helmet. *Source:* Mysore Agriculture Department, Leslie C. Coleman Archives, ca. 1928.

Badami was quite accomplished prior to matriculating at Cambridge. In a letter of introduction, Barber (1923, January 9) wrote that “[Badami] was the best student I had when at Coimbatore and has now had eleven years at plant breeding under the Mysore Government in Dr. Coleman's Department of Agriculture.”^3^ Leslie Coleman, a Canadian entomologist, was Director of the Hebbal experimental station in Mysore, where Badami served (see Figure 1).

**Figure 2:**
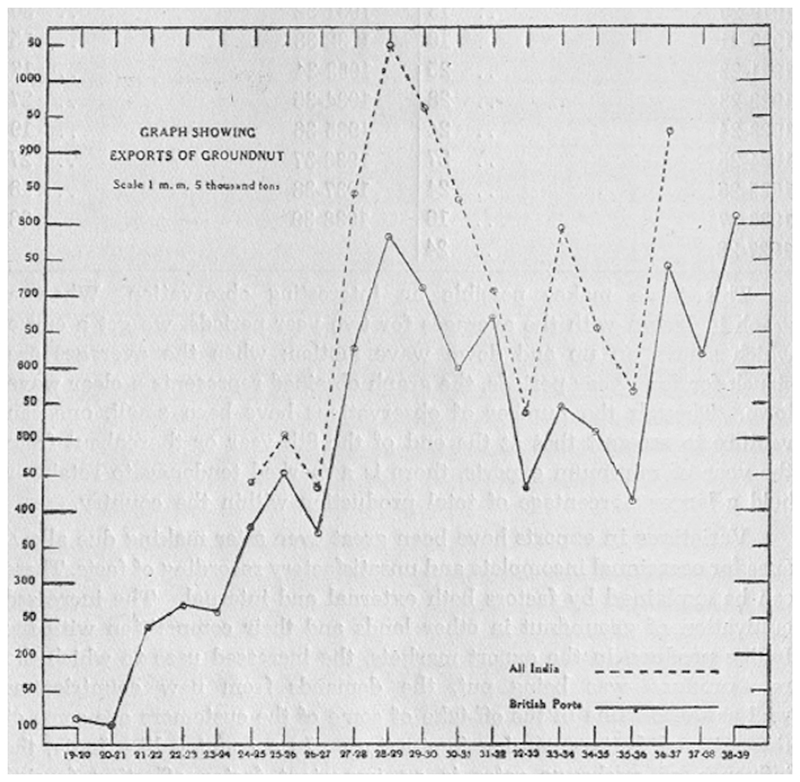
Graph showing groundnut exports from India from 1919 to 1939, starting around 100 t and peaking at over 1000 t in 1928–1929. Notice that British ports comprise the majority of groundnut exports for all of India (Naidu & Hariharan, 1941, p. 86).

It was Coleman who issued the approval to send Badami to Cambridge, yet Badami's history with Barber proved influential in the enrollment process. Barber (1923, January 9) acknowledged that Badami “has had a distinguished career in Mysore and has devoted much time to successful breeding of sugar cane, ragi (*Eleusene coraoana*), potatoes, and ground nut (*Arachis hypogaea*).” The latter crop held great promise, gaining popularity as an export crop in South India (see Figure 2). Groundnuts fit into the rotation with ragi (millet), increasing yields from some of the poorest soils (Baker, 1984).

**Figure 3:**
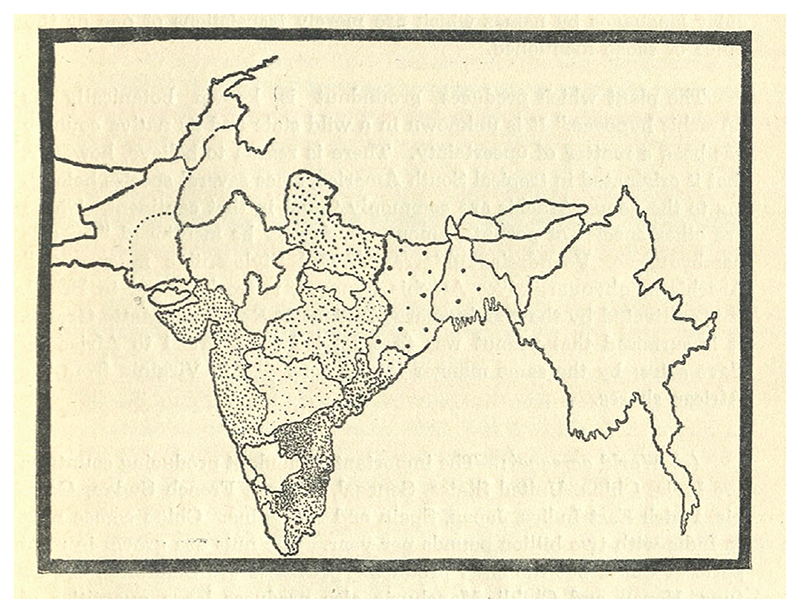
Acreage planted to groundnut into 1930s. The greater density of black dots indicates more acres under groundnut cultivation. Mysore is blank, landlocked in the lower middle of the map, for lack of data. The Madras Presidency to the east, and its Coromandel coast bordering the Indian Ocean, had the most land devoted to groundnuts (Naidu & Hariharan, 1941, p. 9).

**Figure 4:**
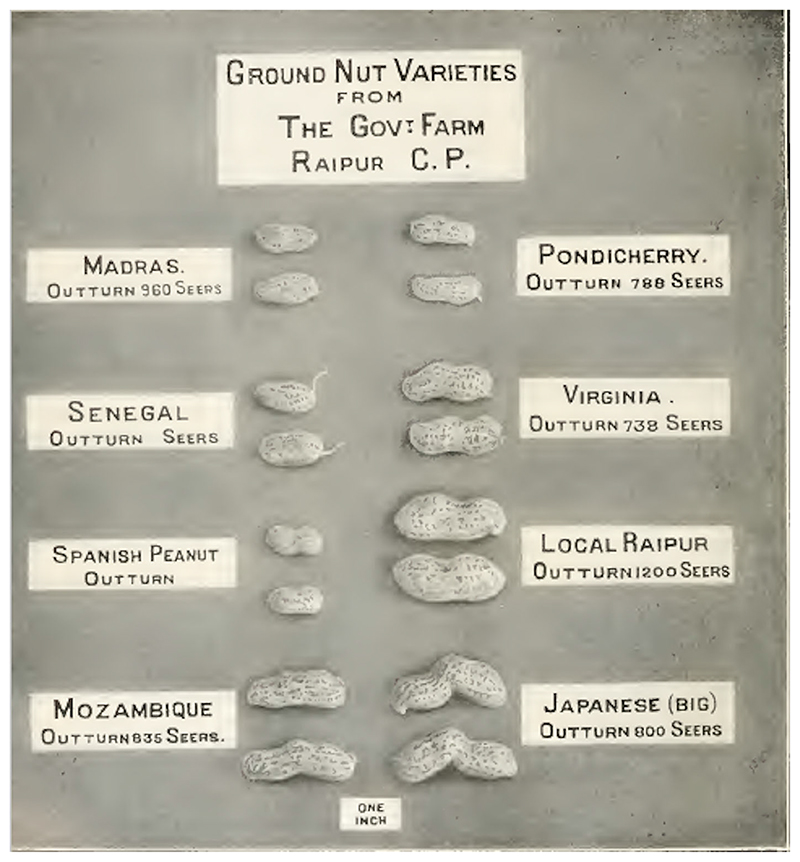
Comparative sample of groundnut varieties at a government farm in Central Provinces, India (Clouston, 1910, plate IX).

Groundnut research in South India during the late 1920s focused on developing a short-season variety that could squeeze into the annual production cycle. While the neighboring Presidency of Madras was, by far, the premier groundnut-growing region in South India, acreage planted to groundnuts in Mysore increased manyfold after the First World War (see Figure 3). The expansion could be attributed not to irrigation but the reopening of postwar markets overseas and the introduction of short-season varieties (Badami, 1938a). Like his peers elsewhere in India, Badami secured a large collection of groundnut varieties to enter into field studies and breeding trails (see Figure 4).

The policy of Mysore, as with the British empire in general, was to develop commercial groundnut varieties for the export economy. All groundnuts can be divided into two basic types based on growth habit. Oil mills in Marseilles, the major port city of the groundnut trade, began to demand nuts from “bush-type” varieties because these groundnuts produced more oil than “spreading” varieties; however, the bush groundnuts were lower yielders, so the Indian cultivators (ryots) suffered financially by adopting the type. Plus, because ryots in South India took annual loans against the upcoming harvest, a failed crop spelled financial doom. Badami (1930b) understood the potential consequences of a cash crop ill-suited to the region, and he insisted that “any improvement in yield of oil content should be combined with the early habit of growth,” to produce a short-season bunch groundnut that ripened in time to beat the onset of drought.

According to scientists in India, an introduction of groundnut seed by the merchant trader Parry & Company had saved local farmers from crop disease and varietal exhaustion at the end of the 19th century (Naidu & Hariharan, 1941). Others, however, suggest that the foreign seed was responsible for the disease (Selladurai, 2018). The introduced groundnut was a spreading type called “Mauritius,” coming to India from that island yet having originated in Mozambique. This variety became the local standard to such an extent that it became called “Coromandel” on the market, renamed after this coastal region of the Indian subcontinent. The introduction was followed by other groundnuts from Japan and the United States at the turn of the 20th century.

**Figure 5:**
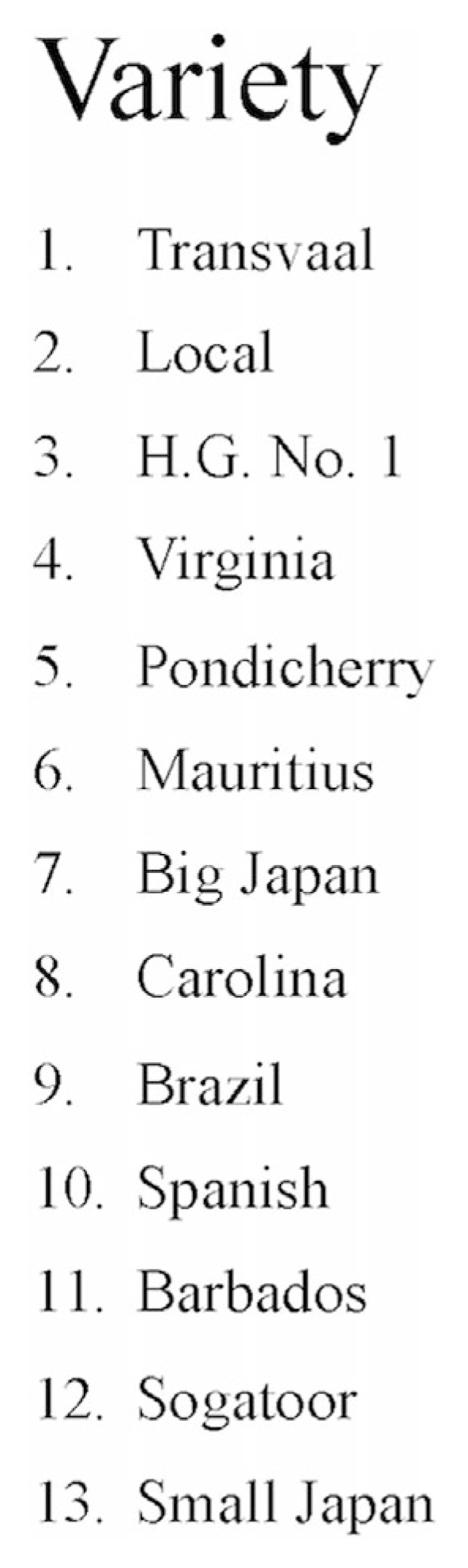
List of groundnut varieties trialed by Badami in Mysore for 1926–1927. His reports from the experiments include average number of pods per plant, percentage of one-, two-, and three-seeded pods, and yield per acre (Badami, 1930b, p. 177).

Under Badami, the Mysore Department of Agriculture entered these groundnuts alongside local varieties in experimental field trials (see Figure 5). In the 1920s, the term “segregates” was used to refer to plants derived from hybrid parentage that displayed specific characters. Breeders conducted field trials to find out which segregates could be line-bred to produce a stable, homozygous variety. Badami, prior to relocating to Cambridge, began experimental crossbreeding with groundnuts at the Hebbal agricultural school, studying the uniformity of number, shape, size, and vigor of seed. His objective was to produce scientific knowledge and to breed a better groundnut for South India.

The commercial growth of the groundnut crop in South India helps explain why Badami left to study under Professor Biffen in Cambridge. Still, the Mysore Department of Agriculture agreed to grant Badami's leave with a certain reluctance. Coleman (1923, December 18), in a letter to the university, invoked a specific request to reduce the course of Badami's study by three terms, out of the ordinary six. Biffen (1924, January 18) responded to the registrar in no uncertain terms, stating that despite Badami's experience, allowing less than 2 years in residence was unwise: “The most he can do is to make crosses and observe the results of the first-generation hybrids. Unless something altogether unforeseen turns up, this would give him an almost negligible amount of material for a thesis.” The Degree Committee agreed that it was impossible to produce a satisfactory thesis in half the normal time (Wood, 1924, February 20). Ultimately, Coleman's appeal for a special allowance resulted in a 1-year reduction for Badami's coursework. His studies were squeezed for time because of official duties to the Mysore Department of Agriculture.

## 3 Off to Cambridge

By January 1924, Badami had a mailing address in Cambridge. What is more, he had brought groundnut seeds from Mysore to continue his breeding work (Badami, 1924, January 14). The field of plant genetics developed through seed from near and far, although the materials were rarely accompanied by the breeders themselves. One year into his studies, Biffen expressed satisfaction with Badami's progress. The courses attended, laboratory work underway, and institutions visited amounted to an impressive list. Among the accomplishments, Badami had “worked in the library for 15 days” at Kew Gardens, participated in the “Empire Exhibition at Wembley for a month,” attended the Royal Agricultural Show at Leicester, and visited the John Innes Horticultural Institute and Rothamstead Experimental Station (Biffen, 1925a, March 4). He even found time to tour experiment stations and seed farms in Denmark and Sweden. Badami was making the most of his overseas experience.^4^

Apparently, his thesis suffered because of it. Praised as an exemplary student in India, Badami's empiricism was suddenly subjected to doubt in Cambridge. In November 1925, a member of his committee, F.L. Engledow, stated, “Barber & I have read V.K. Badami's thesis & are of [the] opinion it is poor,” and he instructed Badami to bring a microscope to his office to check “the merit of his actual slides” (Engledow, 1925a, November 28). Neither the results of Engledow’s review or what prompted it are known, other than the time-honoured tradition of academic gatekeeping.

Whatever misgivings accompanied the research, Badami stayed the course. At the end of the 2-year residency, Biffen wrote to the Government of Mysore and complimented Badami for his industriousness. The professor indicated that, at his instruction, the student had “widened the scope of his programme” because the initial research plan experienced a few setbacks (Biffen, 1925b, December 14). Badami had

attempted to push the study of the genetics of the [groundnut] crop further, using for this purpose material which he brought with him from India. Unfortunately, climatic conditions here [in England] proved unsuitable for growing earthnuts satisfactory [sic] and the work was badly handicapped from the outset.

(Biffen, 1925b, December 14)

In response to these circumstances, Biffen encouraged Badami to make use of the facilities of the National Institute of Agricultural Botany to learn the latest methods of seed testing.

Barber, too, produced a report on Badami's progress for the Government of Mysore. He wrote:

I have formed a very high opinion of his intelligence and anxiety to profit in every way by his stay at Cambridge. He is an ideal student … He is the type of student and researcher which we most prize; and I only wish that it were possible for him to remain permanently in Cambridge, when it would be safe to prophesy a very successful career.

(Barber, 1925a)

As it was, the Mysore Government had granted Badami permission for an expedited leave only. Staying in Cambridge was out of the question.

Barber and Engledow both read Badami's thesis prior to his return to Mysore in December 1925. His academic work was found wanting in comparison with the appraisal of his person. Barber (1925b) stated that “there is nothing original” in most of the thesis, even though the student had “collected practically everything that is known at present about the plant.” He believed that its generality and bulk of congested facts could be explained by haste. Barber's opinion was that Badami deserved an M.Sc. degree.

Similarly, Engledow found Badami difficult to examine. The thesis, despite its records and tables, showed “no attempt at analysis,” which led Engledow (1925b, December 3) to the conclusion that “its results cannot properly be ascribed to scientific method.” These comments among colleagues indicate the extent to which scientific findings were increasingly expected to adhere to a statistical format (Parolini, 2015). Engledow determined that Badami's result was below the M.Sc. standard. Nonetheless, he thought the student ought to be granted an extra year for revisions, followed by a written examination covering general topics in genetics. Biffen agreed to a postponement. The Degree Committee advised Badami to revise his thesis once he obtained more results in India (Wood, 1925, December 7).

Before his departure home, Badami submitted a proposal for a hybridization scheme to undertake in Mysore. The work included crossing the groundnut varieties Hebbal Hybrid and Small Japan, “to get an early maturing strain like Hebbal Hybrid but with high oil contents,” as well as a 12-variety cytological study on the number of chromosomes in *Arachis* (Badami, 1925). Biffen endorsed the ambitious research plan.

## 4 Some Challenges of Going Home

Throughout 1926, Badami was burdened by the workload in Mysore and a prolonged drought. “It has been very dry this year and the monsoon has been a failure in these parts,” he wrote, a failure which delayed his research. The expected timeline for completing his Ph.D. seemed increasingly untenable. Plus, as Badami (1926, August 19) explained, “there has been a complete change in the Gov't of Mysore” with consequences for the agriculture department, including Badami's advancement, which was withheld “pending my securing the Ph.D.” The stakes for completion were easy to understand.

In Mysore, Badami isolated a large number of segregates, the goal being to fix or stabilize a variety with desirable traits. This practice can be observed in the Hebbal Groundnut No. 1 (H.G.1). The variety H.G.1 began in 1921 as “a single bushy plant” that Badami found in a plot of spreading Big Japan. The following year, four seeds were planted, with a Mendelian ratio appearing in the offspring—one plant stood upright, and the other three spread. According to Badami, “Whenever the erect plants were isolated and grown they produced erect plants only,” and these became H.G.1 (Badami, 1930b). The selection had big pods with high oil content and resistance to leaf spot disease.

The variety H.G.1 was distributed by the Mysore Department of Agriculture and immediately became quite popular as it out-yielded the local standard, Mauritius. Badami (1930b) noted that “the pods of this [Hebbal] strain have all the commercial qualities required by the trade.” The groundnut had one major drawback, however. While H.G.1 matured faster than Mauritius, its nuts ripened 2 weeks later than other bunch varieties and failed to fully develop under the physical stress of early drought. This observation anticipated a move away from H.G.1.

After some months apart, Biffen (1926, August 31) expressed concern about his student's pending deadline, suggesting that Badami eliminate aspects of the study. The student's cytological study remained of interest to the professor because of its findings. A small-seeded hybrid had exhibited unexpected chromosome behavior, with significant theoretical implications for future work. Haploid counts by Badami would enter into subsequent studies that sought to answer the question of *Arachis* origins (Husted, 1936). This was precisely the kind of data appreciated within academic circles. Whereas H.G.1 may have served ryots' interests and that of industry in Marseilles, the theoretical objectives of plant genetics were less self-evident.

Badami sent a thesis draft to Biffen in March the following year and expected to submit in full before October. The deadline came and went. Again, “the abnormal seasonal conditions have delayed the ripening of pods,” Badami (1927, October 20) explained, interrupting his plan to study second-generation hybrids. What the Degree Committee had feared was coming to pass. Badami asked to delay the deadline, this time for personal reasons. “Owing to heavy work in the Dep’t in addition to work on Arachis my health has broken down a little,” he admitted (Badami, 1927, October 20). Following 2 months of influenza, Badami (1928, February 16) was able to continue his work, hopeful to dispatch the thesis in April 1928. The Degree Committee began to receive installments of the thesis that July (Priestley, 1928, July 24).

In its final format, Badami's (1929) massive doctoral thesis looked at the historical geography of *Arachis* diversity, the hereditary factors in groundnuts, the hybrid breeding work in Mysore, and more. Two examiners decided in November that “there is a prima facie case for awarding him the Ph.D. degree” (Adie, 1928, November 15). But upon review of Badami's work, Engledow could only recommend an M.Sc. degree. “I have had great difficulty in reaching a decision upon the merits of Mr. Badami's work,” he confessed, because Badami had opted for “the free-range [approach] of the pioneer” (Engledow, 1928). The study succeeded in establishing the mode of inheritance for several characters in *Arachis*, yet Engledow was disappointed by its lack of statistical analysis. He put the final mark below the standard for a Ph.D.

Biffen adopted a different perspective. In evaluating Badami's thesis, he “tried to approach the subject from the point of view of one man whose main object is to improve the crop primarily for the benefit of the Indian agriculturist” (Biffen, 1928, December 7). This remark explains why Badami's study emphasized botany and breeding. Biffen (1928, December 7) reported that “the scope and nature of the thesis are so different from those usually submitted for the Ph.D. degree that it is impossible to compare it” with that of other candidates. While Biffen agreed that the work deserved criticism, and took some responsibility for it, he felt it best to encourage Badami's less specialized style. The student may have entered into the programme without a well-defined research plan, but as Biffen saw it, that was not the same as indecisiveness. Badami was working with ryots back home in mind. His Ph.D. committee wanted mathematical explanations, yet for Biffen, scientific knowledge of genetics was also signified by an ability to describe and produce useful plants. Badami did his best to satisfy academic requirements while simultaneously employing systematic breeding for the problems ryots faced.

## 5 Sacred Finish

Two years had passed since Badami left Cambridge when, in January 1929, he sat in the Mysore office of Leslie Coleman for a 5 h exam. (The mills of Marseilles could not apply greater pressure.) The administered exam was a review of general genetics as required from earlier readings of his thesis. The committee was delighted with the results and with Badami's knowledge of *A. hypogaea.* Conferral of the research degree awaited one additional read by Dr. Martin Leake, a prominent plant geneticist and agricultural economist.

Consistent with earlier remarks, Leake (1929, May 29) praised the industry of the literature review but found Badami's scientific experiments wanting:

His account of his breeding work should indicate that he is using as parents plants known to be genetically pure. I can find nothing to indicate that he recognized this point. His numerical determinations of qualitative characters appear to me forced to fit a preconceived constitution.

Consequently, Leake judged the study undeserving of a Ph.D.

The conceptual coercion noted by Leake was exemplified by Badami's reference to genetic linkage in the origin of H.G.1. The reviewer understood the groundnut to be a natural cross, nothing more (Leake, 1929, May 29). Years before, Badami (1922) had described the same hybrid as a “natural cross,” so why he would have offered a contrary hypothesis at this point is unclear. Leake ultimately sided with Engledow. Both felt that the work did not meet the standard required for a Ph.D., only an M.Sc. degree (Leake, 1929, June 5). The final decision was put to a vote. Six of the 10 members motioned to award the Ph.D., with the other 4 abstaining. Biffen's opinion ruled the day.

Biffen was subsequently asked to attend the Board of Research Studies meeting in Cambridge to explain the outcome and its unusual circumstances. When that day came, he wrote: “Don't expect me at the Research Degrees meeting this afternoon. I'm just rushing off into the fen to show a blinking Yankee how to grow crops” (Biffen, 1929). The revised thesis was approved in June 1929 with a Ph.D. degree conferred by proxy the following year, at which time Badami had become the Economic Botanist in Mysore. “To an Indian finishing a task undertaken is a sacred duty and I am glad I have done my duty,” he told the registrar (Badami, 1930a).

Cambridge provided Badami with an opportunity to study genetics and meet with European plant scientists. By the early 1930s, leading agricultural scholars from the United States and Great Britain, including Leake, were citing Badami's findings on groundnuts (Hunter & Leake, 1933; Stokes & Hull, 1930). Ryots in South India gave their own show of support. The hybrid groundnut from Hebbal gained popularity, even in neighboring Madras. Badami continued with his breeding work to address the shortcomings of H.G.1, amassing 434 segregates by 1931.^5^ Over a decade of sustained research placed Mysore at the forefront of groundnut breeding in South India.

Historian Roll-Hansen (2000) insisted that the entire debate about Mendelism and breeding failed to consider the role of theory in setting research objectives. In his opinion, scholars looking for the utility of genetics in agriculture before 1930 expect a preposterous immediacy, as if the purpose of scientific knowledge was solely to serve industry. The educational experience of V.K. Badami suggests another aspect of this division between genetic theory and practical plant breeding. The commitments of academic genetics reflected a geography of power. Observations from princely states were valued for the “heady” theory occurring at places like Cambridge (Ratcliff, 2016). Within the context of empire, agricultural research prioritized economic botany and the increase of commodity crop exports. Seasonal workloads restricted the time available for abstractions. For Indian agricultural officers, this was true even when enrolled at university.

A Ph.D. from Cambridge gave Badami the credentials to advance his career in government, although his duties to the Mysore Department of Agriculture nearly obstructed the successful completion of his thesis. He would become the Principal of the Agricultural School in Hebbal and, in 1934, the Deputy Director of Agriculture for Mysore. As with the women who helped William Bateson establish the discipline of genetics (Richmond, 2001), Badami's contribution to science awaits a fuller accounting.

## Data Availability

Data sharing is not applicable to this article as no datasets were generated or analyzed during the current study.

## Footnotes

1 Certain online sources use the name Venkatrao K. Badami. The spelling fits his abbreviated authorial title, but lacks support from any publication, including his thesis.

2 A short biography of V.K. Badami and his father, Badami Krishna Rao, was included in the Silver Jubilee Souvenir of the Mahratta Education Fund (1937), under Eminent Maharashtrians of South India. p. 112,121.

3 All letters cited are from Badami's student file (1923–1930) at the University of Cambridge Archives (UA BOGS11920-37/73).

4 This extracurricular pedagogy provides a useful comparison with experiences at agricultural research institutions in the United States, as detailed by Kimmelman (1987).

5 The large-seeded Hebbal Groundnut No. 2, derived from a cross between Small Japan and Virginia, was the next variety in the Hebbal line (Badami, 1938b).
